# Do floral and ecogeographic isolation allow the co‐occurrence of two ecotypes of *Anacamptis papilionacea* (Orchidaceae)?

**DOI:** 10.1002/ece3.7432

**Published:** 2021-07-02

**Authors:** Salvatore Cozzolino, Giovanni Scopece, Michele Lussu, Pierluigi Cortis, Florian P. Schiestl

**Affiliations:** ^1^ Department of Biology University Federico II of Naples Napoli Italy; ^2^ Department of Life and Environmental Sciences University of Cagliari Cagliari Italy; ^3^ Istituto Regionale per la Floricoltura (IRF) Sanremo Italy; ^4^ Department of Systematic and Evolutionary Botany and Botanical Gardens University of Zurich Zurich Switzerland

**Keywords:** deceptive pollination, ecogeographic isolation, floral isolation, floral traits, food deception, orchids, premating barriers, sexual deception

## Abstract

Ecotypes are relatively frequent in flowering plants and considered central in ecological speciation as local adaptation can promote the insurgence of reproductive isolation. Without geographic isolation, gene flow usually homogenizes the allopatrically generated phenotypic and ecological divergences, unless other forms of reproductive isolation keep them separated. Here, we investigated two orchid ecotypes with marked phenotypic floral divergence that coexist in contact zones. We found that the two ecotypes show different ecological habitat preferences with one being more climatically restricted than the other. The ecotypes remain clearly morphologically differentiated both in allopatry and in sympatry and differed in diverse floral traits. Despite only slightly different flowering times, the two ecotypes achieved floral isolation thanks to different pollination strategies. We found that both ecotypes attract a wide range of insects, but the ratio of male/female attracted by the two ecotypes was significantly different, with one ecotype mainly attracts male pollinators, while the other mainly attracts female pollinators. As a potential consequence, the two ecotypes show different pollen transfer efficiency. Experimental plots with pollen staining showed a higher proportion of intra‐ than interecotype movements confirming floral isolation between ecotypes in sympatry while crossing experiments excluded evident postmating barriers. Even if not completely halting the interecotypes pollen flow in sympatry, such incipient switch in pollination strategy between ecotypes may represent a first step on the path toward evolution of sexual mimicry in Orchidinae.

## Graphical abstract

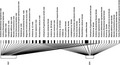

## 1 INTRODUCTION

Adaptation to different environmental conditions or habitats may promote the evolution of genetically different forms of a species, that is, ecotypes (Turesson, 1922). The process is relatively frequent in flowering plants with wide distribution and its role in plant speciation has been widely recognized, as ecotypes may represent a first step in the accumulation of reproductive isolation along the so‐called speciation *continuum* (Lowry & Gould, 2016; Nosil, 2012). Typically, different ecotypes are adapted to different ecological conditions hence geographic isolation is the main barrier for preventing their meeting and, eventually, intermixing (Baack et al., 2015). However, when different ecotypes come into secondary contact and/or occur in proximate/geographically close habitat that may allow a large amount of gene flow among ecotypes, introgression and admixture, rather than reinforcement of phenotypic divergence, are the most likely outcomes (Sancho et al., 2018; Zitari et al., 2012). This process is considered a reversal along the speciation *continuum* and is among the main causes of loss of allopatrically acquired biodiversity (Seehausen et al., 2008).

Adaptation to different habitats may also drive the evolution of partially reproductively isolated ecotypes that can persist in sympatry or parapatry despite some gene flow. This is often due to ecologically based prezygotic mechanisms that can involve, for instance, flowering time or pollination strategy (Briscoe Runquist et al., 2014; Lowry et al., 2008). Plant species with wide geographic ranges may experience different pollinator set (Johnson, 2006, 2010; Van der Niet et al., 2014), varying in absolute or relative pollinator composition. This may lead to a different strength and direction of the pollinator‐mediated selection and, consequently, to a geographic variation in floral traits subject of selective pressure (Anderson et al., 2010; Newman et al., 2014). Indeed, local adaptation of ecotypes to different pollinator species can initiate speciation (Van der Niet & Johnson, 2009; Sobel & Streisfeld, 2015). In this circumstance, recently diverged lineages can have accumulated local adaptation to different pollinators that impedes or slow down their intermixing, hence, an intraspecific polymorphism can be established with the coexistence of two or more morphs phenotypically differentiated (Leimar, 2005). Partial reproductive isolation between ecotypes can help the maintenance of genetic differences in sympatry as long as there is strong pollinator‐mediated divergent selection that overwhelm the presence of some ongoing gene flow (Rymer et al., 2010). Accordingly, most of the studies that have examined transition between different ecotypes of the same plant species highlighted the importance of pollinator‐assortative mating (Anderson et al., 2010; Newman et al., 2014). At the same time and as expected within species, the presence of postpollination/postzygotic barriers has only been found between ecotypes that were also differing in ploidy level (i.e., cytotypes; Pegoraro et al., 2016; Husband & Sabara, 2004; but see Richards & Ortiz‐Barrientos, 2016 for some notable exceptions).

Here, we investigated the factors that allow the maintenance of phenotypic divergence in the sympatric orchid ecotypes *A. papilionacea* subsp. *papilionacea* and *A. papilionacea* subsp. *grandiflora* (hereafter referred to as *A. p. papilionacea* and *A. p. grandiflora*, respectively).

The pollination strategy of *A. p. papilionacea* has been investigated in previous studies that found an important contribution of male hymenopterans of different species (Scopece et al., 2009; Vogel, 1972; Vöth, 1989). Dressler (1981) suggested that this strategy of male insect attraction could represent the first step in the evolution of sexual mimicry. The pollination of *A. p. grandiflora* remains fairly little known but the two ecotypes show evident phenotypic differences that suggest different pollination strategies. In particular, several floral clues and the lack of nectar (true for both ecotypes) indicate a generalized food‐deceptive strategy for *A. p. grandiflora*, commonly found among other members of the genus *Anacamptis* (Van der Cingel, 1995). Accordingly, and differently from *A. p. papilionacea*, *A. p. grandiflora* shows a large flattened labellum and prominent nectar guides that are typical flower traits involved in generalized food deception (Johnson & Schiestl, 2016). While pollination by sexual mimicry relies solely on male pollinators, food‐deceptive pollination mostly relies on female pollinators, foraging for nectar or pollen reward. Female pollinators also show a very different behavior than males when landing on the flowers (Ne’eman et al., 2006). Thus, the adoption of female versus. male pollinators may enhance assortative mating and produce strong disruptive selection between morphs, for example, on flower morphology and scent. Concordantly, Scopece et al., (2015) found differences in pollen transfer efficiency when examining allopatric populations of *A. p. papilionacea* and *A. p. grandiflora* suggestive of different pollinator behavior.

In this study, we aim to understand how the two *A. papilionacea* ecotypes remain distinct even when coexisting. To fulfill this aim, we addressed the following specific questions:

1. Are there differences in pollination strategy between the two ecotypes?
2. What is the extent of phenotypic differentiation between *A. p. papilionacea* and *A. p. grandiflora* and is it maintained in sympatric populations?
3. Which factors contribute to reproductive isolation?
4. Is there any difference in ploidy level between the two ecotypes?
5. Are there any intrinsic pre‐ and postzygotic barriers that contribute to the maintenance of the two ecotypes?

## 2 MATERIALS AND METHODS

### 2.1 Study system and study areas

**FIGURE 1:**
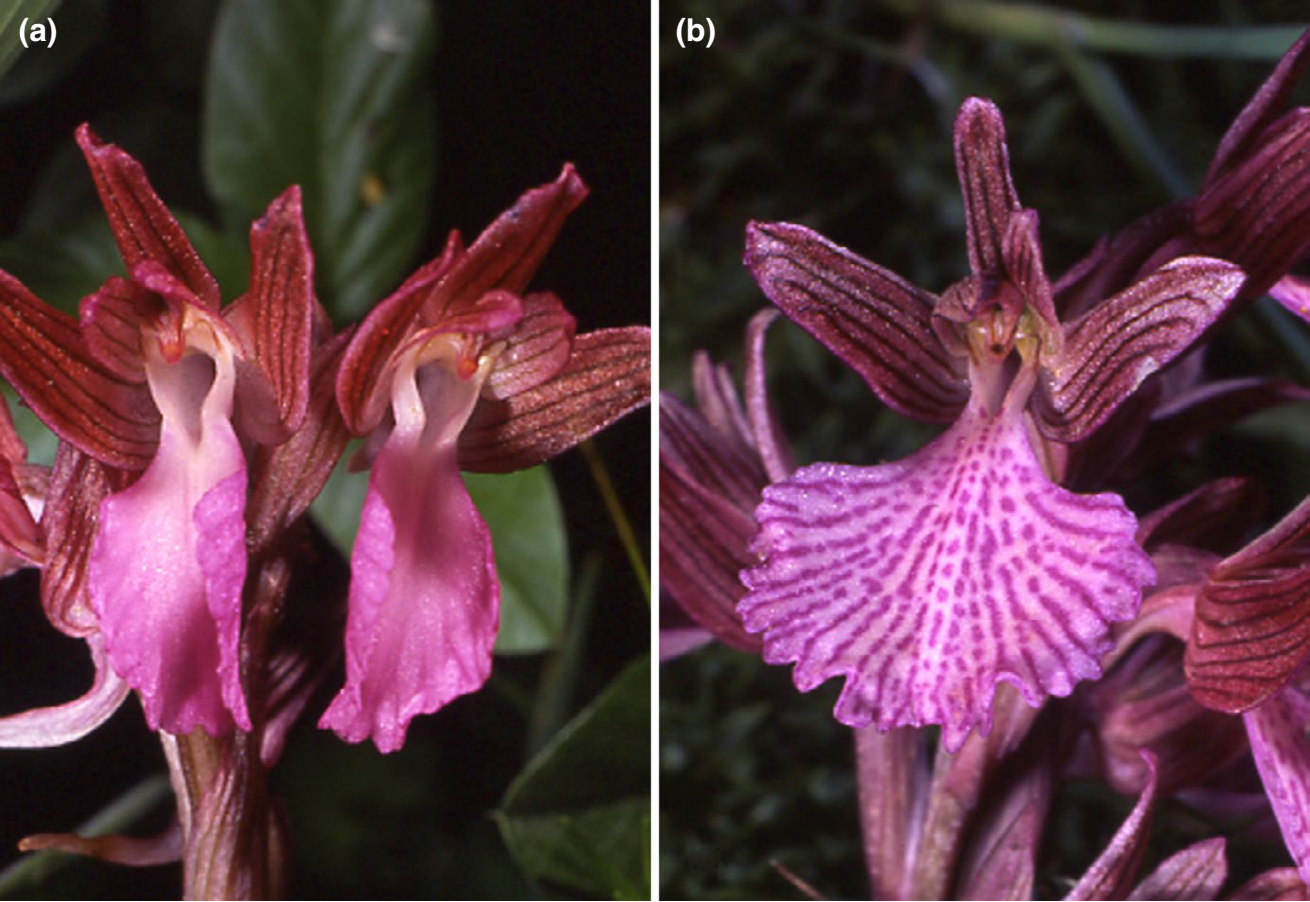
Flowers of the two ecotypes of *Anacamptis papilionacea*: (a) *A. p. papilionacea* and (b) *A. p. grandiflora* (Photographs courtesy of R. Romolini)

*Anacamptis papilionacea* is a Mediterranean orchid within a clade of food‐deceptive species (Aceto et al., 1999). It is self‐compatible but needs insects to transfer the pollen (Scopece et al., ,^2007^, ^2009^). This species contains two most common ecotypes, *A*. *papilionacea* subsp. *papilionacea* and *A. papilionacea* subsp. *grandiflora* (Figure 1), that are mainly allopatric but coexist in some Mediterranean regions. In some of these contact zones (e.g., in Southern Italy; Scopece et al., 2009), *A. papilionacea* populations show a clear prevalence of one ecotype while in others, as on Sardinia island, both ecotypes co‐occur without any detectable genetic differences (Arduino et al., 1995).

*A. p. papilionacea* grows in Mediterranean maquis and rough grasslands in alkaline soils from 0‐ to 1100‐meter altitude. Its main distributional range is the central Mediterranean basin: Corsica, Sardinia, Italy, Croatia, Serbia, Macedonia, Albany, northeastern, and northwestern Greece (Baumann, 2006; Kretzschmar et al., 2007). *A. p. papilionacea* inflorescences narrow toward the top and contain 4–15 flowers with reddish‐violet labellum in the outside part and that tend to disappear in the lucid center. Overall, *A. p. papilionacea* displays some typical floral traits for generalized food deception (as a long spur and a large colored labellum). However, differently from other food‐deceptive species, *A. p. papilionacea* has been found to be primarily pollinated by male hymenopterans, for example, *Eucera tuberculata* males in a population on Elba Island (Vogel, 1972), *Eucera bidentata* males in some Greek populations (Vöth, 1989), *Eucera nigrescens* males (Cozzolino et al., 2005) or *Anthophora crinipes* males (Scopece et al., 2009) in southern Italy. This unusual attractiveness for male hymenopterans suggests pollinator attraction based on some sexual signals and Faegri and Van der Pijl (1979) coined the term of “rendezvous attraction” for this peculiar pollination mechanism. Because volatile signals are key stimuli in the sexual behavior of bees (Kullenberg & Bergström, 1976), the preferential attraction of males by *A. p. papilionacea* suggests that some olfactory signals are likely involved. Schiestl and Cozzolino (2008), by analyzing *A. papilionacea* floral scent, found a prominent production of chemical compounds (alkanes and alkenes) similar to those produced by flowers of sexually deceptive species of the related genus *Ophrys* (Ayasse et al., 2003 *)*, supporting the assumption that chemical signals related to mating behavior may be involved in the attraction of pollinators by *A. p. papilionacea*. Nonetheless, evidence summarized in Van der Cingel (1995) shows that *A. papilionacea* sensu *latu* (i.e., *A. p. papilionacea* and *A. p. grandiflora*) is also pollinated by other insect classes, even butterflies (Vogel, 1972).

The *A. p. grandiflora* ecotype mainly differs from *A. p. papilionacea* by its larger flower size and a very clear pattern of dark reddish‐purple venation (nectar guides) on the whitish/pink labellum. Also, the *A. p. grandiflora* labellum is often larger and flatter than that of *A. p. papilionacea*. In contrast to *A. p. papilionacea*, the pollination system of *A. p. grandiflora* has never been investigated thoroughly, but the presence of marked nectar guides and a large labellum suggests a generalized food deception for this ecotype. *A. p. grandiflora* has habitat requirements like *A. p. papilionacea*, yet with a higher altitudinal range, growing up to 2000m altitude. Its distributional range is western Mediterranean and includes Southern France, Spain, Portugal, Morocco, Algeria, Tunisia, Sicily, and Sardinia, but is less commonly found in southern Italy (Baumann, 2006; Ketzschmar, 2007). The present study was conducted in different allopatric (where a single ecotype was present) and sympatric (where both ecotypes coexist) populations on Sardinia island where both ecotypes are common.

### 2.2 Morphological differentiation

Morphometric analyses were conducted in two sympatric (Campuomu and Magomadas) and two allopatric populations (Porto Alabe for *A. p. papilionacea* and San Michele for *A. p. grandiflora*). For each individual included in morphometric analysis, inflorescence height was measured, the number of flowers counted, and two flowers were picked and stored in EtOH 70%. To obtain floral trait measurements, sampled flowers were dissected, and floral parts were placed between two transparent plastic film sheets. These sheets were subsequently scanned to obtain digital images in a 300 dpi TIFF format with a coordinate millimeter paper on the back for reference; measures of floral traits were later obtained using ImageJ 1.33 software (Rasband, National Institutes of Health, USA). We measured labellum width and length, internal, external tepals width and length, spur length, and length of the bract. Phenotypic traits were compared between *A. p. papilionacea* and *A. p. grandiflora* through a Mann–Whitney U Test. Three independent principal component analyses (PCAs) were performed to explore variation between *A. p. papilionacea* and *A. p. grandiflora* in the allopatric and in two sympatric populations.

### 2.3 Ploidy level

*A. papilionacea* chromosome number was investigated in other Southern Italian populations by D’Emerico et al. (2001) that concordantly showed 2n = 32. Here, we investigated potential difference in ploidy levels between *A. p. papilionacea* and *A. p. grandiflora* by using flow cytometry. In total, nine *A. p. papilionacea* and nine *A. p. grandiflora* samples were analyzed. Two pollinia of a single flower per individual were collected from the sympatric population of Magomadas. For sample preparation and analysis, we followed a two‐step protocol (Dolezel et al., 2007) as described in Xu et al., (2011). The two pollinia were chopped and mashed together with approximately 25 mm^2^ leaf material of *Phaseolus coccineus* (2n, 1C = 1.01 ± 0.4 pg; Bennett & Leitch, 2005) which served as internal standard (IS). The data were processed by using the ratio of integrated peaks of the study organisms and *P. coccineus*.

### 2.4 Pollinators and floral traits

To characterize the pollination strategies of *A. p. papilionacea* and *A. p. grandiflora* in Sardinia, we identified floral visitors, analyzed floral scent and quantified pollen transfer efficiency, a parameter that has been reported to be characteristic for different deceptive pollination strategies (Scopece et al., 2010). Insect capture and identification were performed in 2011 and 2017 in sympatric and allopatric populations of *A. p. papilionacea* and *A. p. grandiflora*. We selected patches in which either *A. p. papilionacea* or *A. p. grandiflora* were the only blooming orchid in order to ensure that insects carrying pollinia were their visitors. Insects were caught with a butterfly net while visiting *A. p. papilionacea* or *A. p. grandiflora*. In allopatric populations, we also caught insects while foraging on nectar plants in the study area and killed those carrying orchid pollinia (the *A. p. papilionacea* and *A. p. grandiflora* pollinia are reddish‐violet and can be easily distinguished from pollinia from other, coflowering orchid species) using diethyl ether and stored them for subsequent identification. This approach, even if allowing to increase the numbers of collected insects has the limitation that does not identify legitimate pollinators (i.e., those transferring pollen between flowers) but only visitors that remove pollinia from the flower.

For floral scent, we focused on low‐volatile cuticular hydrocarbons, because these are the known pseudopheromones used for male‐pollinator attraction in the genus *Ophrys* (Johnson & Schiestl, 2016). Scent was extracted from 20 individuals of *A. p. papilionacea* and 20 individuals of *A. p. grandiflora* in 2011 from the sympatric population of Magomadas. Scent extraction was conducted by picking the labellum from the flower and dipping it for 30 s in 0.2 ml of hexane and removing it thereafter (Schiestl & Cozzolino, 2008). The analysis of the scent and identification of compounds was done using a gas chromatograph (GC) with FID detector and DB‐5 column, following previous studies (Schiestl et al., 2000; Schiestl & Cozzolino, 2008; Schiestl & Marion‐Poll, 2002). Compounds were identified based on few samples being analyzed with GC with mass selective detection and comparison of mass spectra and retention times of synthetic standards and compounds in natural samples. Absolute amounts of compounds in the samples were calculated by using an internal standard that was added before the analysis.

Double bond position in alkenes was determined using synthetic alkene standards. Alkenes with different double bond positions have different retention times on the DB‐5 column used in this study, thus double bond position can be inferred by comparing retention times of natural compounds and synthetic standards (Mant et al., 2005). The cis/trans configuration was not analyzed, but the cis‐configuration was inferred because of its more common occurrence in unsaturated fatty acids, the precursors in the biosynthesis of alkenes (Schlüter et al., 2011).

Data were analyzed statistically through a hierarchical clustering on principal component (HCPC) analysis using the R package *FactoMineR*. Five outlier individuals of *A. p. grandiflora* were removed from the analysis. The relative amounts of compounds (in %) were also calculated and compared by one‐way ANOVA with significance level set to 0.01 (Bonferroni correction).

To estimate pollen transfer efficiency, flowers of every selected plant were checked for pollen removal and pollen deposition. This observation was performed in the field with a 10x magnification lens in the sympatric population of San Priamo. The efficiency was quantified as the ratio between flowers pollinated (with at least one pollen massula in the stigma) and flowers visited (i.e., that exported at least one pollinium). Standard errors for pollen transfer efficiency values in the two ecotypes were obtained with 1,000 bootstraps.

### 2.5 Environmental niche modeling (ENM) and estimation of ecogeographic isolation

To model the environmental niche of *A. p. papilionacea* and *A. p. grandiflora*, we used MAXENT 3.4.0 (Phillips et al., 2006) a software that is suitable for presence‐only data, particularly with smaller sample sizes (<25) (Townsend Peterson et al., 2007; Wisz et al., 2008). To construct the ENMs, we selected 19 abiotic variables from the Worldclim 2.1 database at the 2.5 arc‐min (~4 km^2^) resolution (Kantar et al., 2015). Climatic data are derived from temperature and rainfall annual trends between 1970 and 2000. Form the whole variables, we excluded those showing levels of correlation higher than 0.7. We thus selected seven variables (Table 1). With these data, we first estimated the environmental niche of *A. p. papilionacea* and *A. p. grandiflora* in Sardinia and then their overlap. To estimate niche overlap, we used the Schoener's 1968, that is, the joint probability density function of the multidimensional niche indicators at alpha = 95%. We calculated RI_ecogeography_ as 1‐ % of niche overlap (Warren et al., 2008).

**TABLE 1:** Mean value and coefficient of variation (CV) for the seven climatic variables used in the Environmental Niche Model

Ecotype	Annual mean temperature (Bio 1)		Temperature seasonality (Bio 4)		Max temperature of warmest month (Bio 5)		Min temperature of coldest month (Bio 6)		Annual precipitation (Bio 12)		Precipitation of wettest month (Bio 13)		Precipitation seasonality (Bio 15)	
	Mean (°C)	CV (%)	Mean	CV (%)	Mean (°C)	CV (%)	Mean (°C)	CV (%)	Mean (mm)	CV (%)	Mean (mm)	CV (%)	Mean (CV)	CV (%)
			(sd x 100)											
*A. p. grandiflora*	15.0	11.2	588.8	2.2	28.3	6.1	4.4	24.6	636.5	20.8	844.8	21.0	49.3	3.1
*A. p. papilionacea*	15.7	5.4	595.3	1.8	29.8	4.2	4.9	10.5	528.8	15.8	760.0	18.7	49.2	2.9

### 2.6 Flowering time estimation and calculation of phenological isolation index (RI _phenology_)

Phenological data were recorded in 2012 from the middle of March until the end of the flowering time in the sympatric population of Magomadas. This population was visited periodically and the number of flowering individuals for each ecotype was recorded. Phenological isolation index was calculated as described in Lowry et al., (2008).

### 2.7 Pollen staining experiment and calculation of floral isolation index

In 2011 and 2017, in order to quantify the degree of floral isolation, we built experimental plots with pollen stained of *A. p. papilionacea* and *A. p. grandiflora*. In 2011, in the sympatric population of Magomadas, each plot was built up with four orchid inflorescences (two for each ecotype). Each inflorescence was placed in a vial filled of water into the soil. Within a plot, vials were outdistanced of about 40 cm, forming an outline of a quadratic square. Each plot was then outdistanced of at least 10 m from the next one. We built up four series, each consisting of 10 plots (40 plots). In 2017, to gain a replicate, we performed a series of 10 additional plots in the sympatric population of Santa Sofia (Meana Sardo).

To trace pollen movements within the plots, we stained pollinia of all open flowers of *A. p. papilionacea* and *A. p. grandiflora* with red (Neutral Red) and orange (Orange G) staining, using a 10 ml Hamilton syringe (Peakall, 1989; Xu et al., 2011). Inflorescences were controlled daily with a 10x magnifying glass to detect pollen removal and deposition and were replaced with new inflorescences every five days.

Based on data collected in 2011 and 2017, we calculated the floral isolation index following Sobel and Chen (2014) as: RI_floral_isolation_ = 1 – 2 * (observed interecotype pollen movements / expected interecotype pollen movements) / ((observed interecotype / expected interecotype pollen movements) + (observed intraecotype / expected intraecotype pollen movements)). Following Martin and Willis (2007) and Lowry et al., (2008), expected interecotype and intraecotype pollen flow were both calculated as: interecotype + intraecotype pollen movements / 2. Isolation index was averaged for the two sampling years.

### 2.8 Hand‐pollination experiment and calculation of postmating isolation indices

Hand‐pollination experiments were performed in the Botanic Garden of Cagliari (Sardinia), during the spring of 2017, to estimate postmating isolation indices.

Plants were collected from natural allopatric populations of *A. p. papilionacea* and *A. p. grandiflora* and, to prevent uncontrolled pollinations, were placed in cages covered with a thin nylon net prior to flowering. Ripe fruits, when produced, were collected and stored in silica gel at 4°C. Seeds were subsequently observed under an optical microscope as described in Scopece et al., (2007).

Postmating prezygotic isolation (RI_postm_prezygotic_) was calculated as the proportion of fruits formed following interecotype pollinations, relative to the proportion of fruits formed following intraecotype pollinations within each parental ecotype:

RI_postm_prezygotic_ = 1–2* (% fruit formed in inter‐ecotype crosses / (% fruit formed in inter‐ecotype crosses + % fruit formed in intraecotype crosses)) (McDade & Lundberg, 1982).

Postmating postzygotic isolation (i.e., embryo mortality, RI _embryo_mortality_) was similarly calculated as the proportion of viable seeds obtained in interecotype pollinations, relative to the proportion of viable seeds in intraecotype pollinations within each parental ecotype: Postpostzygotic = 1–2* (% viable seeds in interecotype crosses / (% viable seeds in interecotype crosses + % viable seeds in intraecotype crosses)).

## 3 RESULTS

### 3.1 Morphological differentiation

**FIGURE 2:**
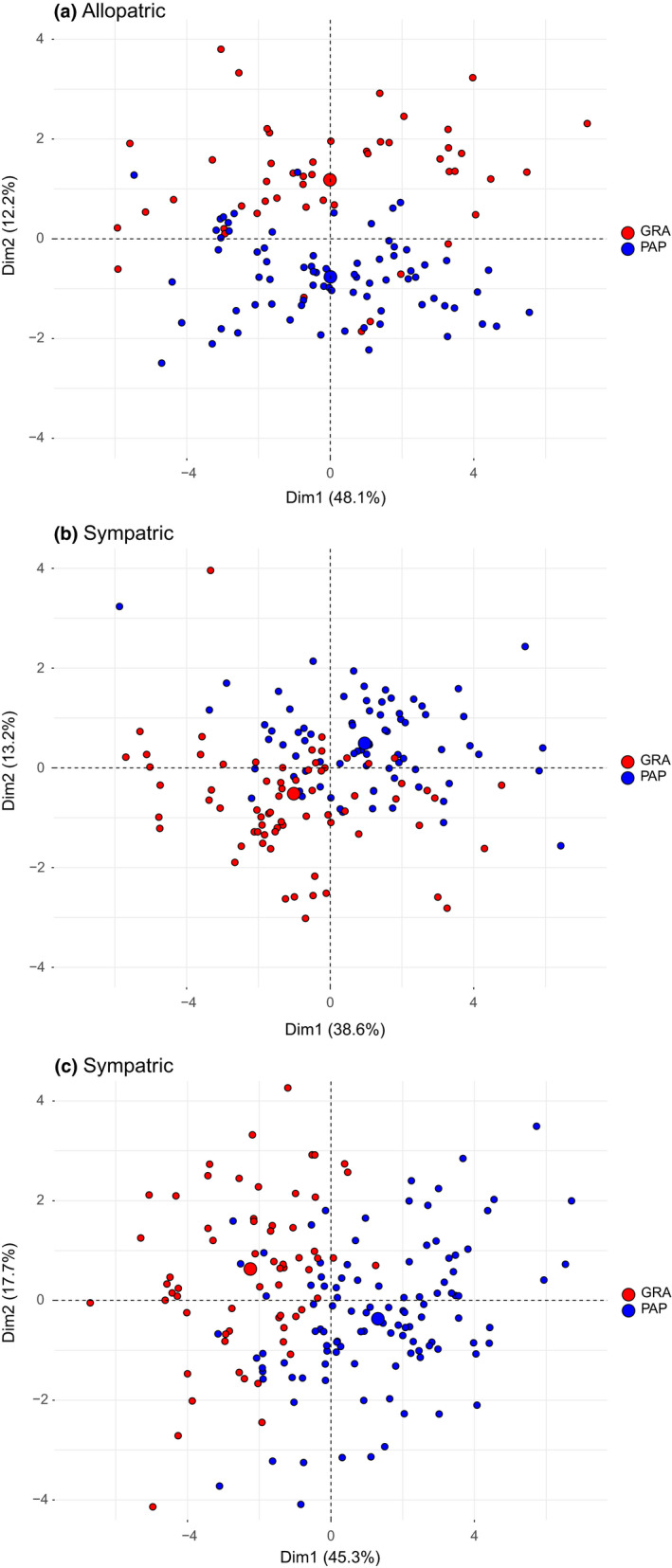
Morphological differentiation between the two *Anacamptis papilionacea* ecotypes (*A. p. papilionacea* and *A. p. grandiflora*). Principal component analyses (PCAs) based on morphological traits for (a) allopatric populations of Porto Alabe (*A. p. papilionacea*) and San Michele (*A. p. grandiflora*), (b) sympatric population of Campuomu, (c) sympatric population of Magomadas. *A. p. papilionacea* in blue, *A. p. grandiflora* in red

*A. p. papilionacea* and *A. p. grandiflora* were clearly morphologically differentiated both in allopatry and in sympatry. In the allopatric populations of Porto Alabe (*A. p. papilionacea*) and San Michele (*A. p. grandiflora*), 8 out of the 11 investigated traits showed significant differences. In the sympatric population of Campuomu, 6 out 12 traits were significantly different. In the sympatric population of Magomadas, 11 out 12 traits were significantly different (Table 2). The three resulting PCAs showed a similar overlap both in sympatric and allopatric populations (Figure 2).

**TABLE 2:** Mann–Whitney U test between phenotypic traits in *A. p. papilionacea* and *A. p. grandiflora*

	Mann–Whitney U test	Labellum width	Labellum length	Right tepal width	Right tepal length	Right sepal width	Right sepal length	Central sepal width	Central sepal length	Spur length	Ovary length	Bract length
Allopatric *A. p. papilionacea* and *A. p. grandiflora*	U	2,239.5	2,430.0	1,291.5	**2,131.0**	**833.5**	**1869.0**	**1,004.0**	**1722.0**	**892.5**	**2,145.5**	2,373.0
	W	5,165.5	5,058.0	3,919.5	**4,759.0**	**3,461.5**	**4,497.0**	**3,632.0**	**4,350.0**	**3,520.5**	**4,773.5**	5,001.0
	Z	−1,905	−1,174	−5,458	**−2,205**	**−7,233**	**−3,220**	**−6,645**	**−3,890**	**−7,072**	**−2,265**	−0.600
	P	0.057	0.240	0.000	**0.027**	**0.000**	**0.001**	**0.000**	**0.000**	**0.000**	**0.023**	0.549
Sympatric population of Campuomu	U	**309.5**	**1,294.5**	1652.5	1658.5	**1,420.0**	1574.5	1818.0	1845.5	**427.0**	**1,093.5**	**1,406.5**
	W	**3,235.5**	**4,220.5**	2,828.5	2,834.5	**4,346.0**	2,799.5	3,043.0	4,771.5	**1652.0**	**2,318.5**	**4,332.5**
	Z	**−7,851**	**−2,870**	−0.880	−0.849	**−2,235**	−1,454	−0.223	−0.083	**−7,257**	**−3,886**	**−2,142**
	P	**0.000**	**0.004**	0.379	0.396	**0.025**	0.146	0.824	0.933	**0.000**	**0.000**	**0.032**
Sympatric population of Magomadas/Noesala	U	**1518.5**	**2,306.0**	3,331.0	**795,0**	**2,509.5**	**815.5**	**2002.5**	**804.0**	**100.0**	**788.5**	**2,771.0**
	W	**7,623.5**	**4,322.0**	5,347.0	**2,811.0**	**4,589.5**	**2,895.5**	**4,018.5**	**2,820.0**	**2,116.0**	**2,804.5**	**4,787.0**
	Z	**−6,141**	**−3,584**	−0.423	**−8,423**	**−3,154**	**−8,441**	**−4,548**	**−8,357**	**−10,616**	**−8,443**	**−2,106**
	P	**0.000**	**0.000**	0.672	**0.000**	**0.002**	**0.000**	**0.000**	**0.000**	**0.000**	**0.000**	**0.035**

In bold significant differences.

### 3.2 Ploidy level

We observed no significant differences in the ratio of internal standard to the ecotypes *A. p. papilionacea* and *A. p. grandiflora* (*t*_3.25,56_ = −0.149; *p* =0.88) indicating that the genome size of the two ecotypes is similar. The ratio was 3.38 ± 0.09 in *A. p. grandiflora* and 3.35 ± 0.06 in *A. p. papilionacea*. This excluded that the two ecotypes differed in ploidy level.

### 3.3 Characterization of pollination strategy

**FIGURE 3:**
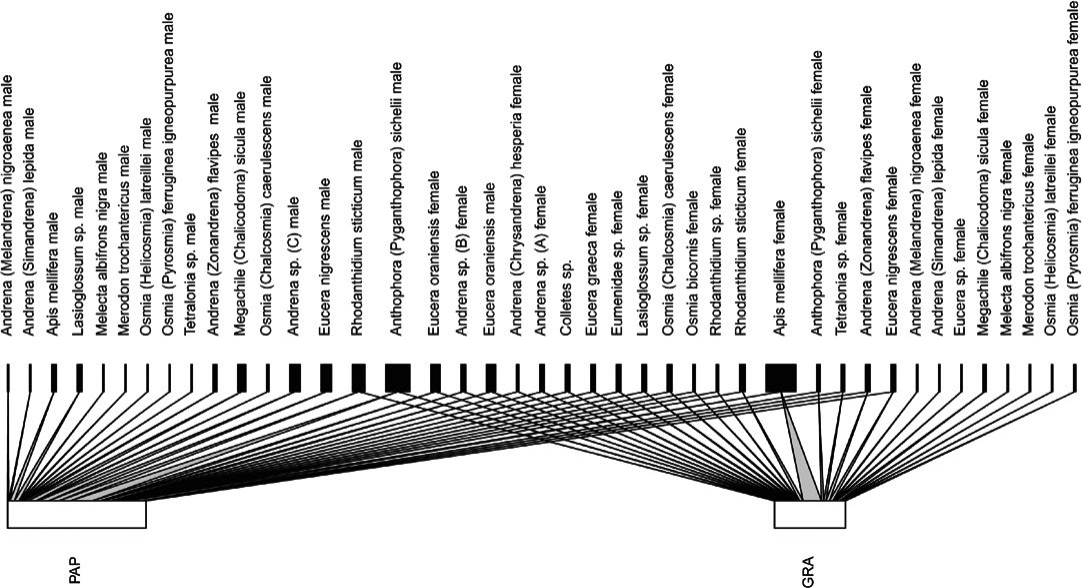
Pollinator sharing between the two *Anacamptis papilionacea* ecotypes. Pollinator network of *A. p. papilionacea (PAP)* and *A. p. grandiflora* (GRA) visualized using the “networklevel” function in the bipartite package68 in R (http://www.R‐project.org)

We collected a total of 183 insects visiting *A. p. papilionacea* or *A. p. grandiflora*, identified all to the generic level and, among them, 136 to the species level. We caught 122 *A. p. papilionacea* visitors (81 males and 41 females), and 61 *A. p. grandiflora* visitors (6 males and 55 females). The ratio of male/female attracted by the two ecotypes was significantly different (Fisher test, *p* <0.0001). The investigated ecotypes attract largely the same pollinator species (25 species *A. p. papilionacea* and 25 species *A. p. grandiflora*) with only two species, one for each ecotype, being exclusive pollinators. The two ecotypes thus showed an intense sharing in terms of insect species, with 24 species pollinating both ecotypes (overall of 96% in *A. p. papilionacea* and *A. p. grandiflora*). However, pollinator sharing decreased when insect sex was considered (Table 3). An important percentage of sharing between the two ecotypes was also due to honeybees: of 76 shared individuals in *A. p. papilionacea*, 10 (13.1%) were honeybees, and of 51 shared individuals in *A. p. grandiflora*, 16 (31.4%) were honeybees. Pollinator sharing was visualized using the “networklevel” function in the bipartite package68 in R (http://www.R‐project.org; Figure 3).

**TABLE 3:** Number and sex of visiting insects collected on *A. p. papilionacea* and *A. p. grandiflora*

Visitor species	Gender	*A. p. grandiflora*	*A. p. papilionacea*
*Andrena (Chrysandrena) hesperia*	F	1	1
*Andrena (Melandrena) nigroaenea*	F	1	0
*Andrena (Melandrena) nigroaenea*	M	0	1
*Andrena (Simandrena) lepida*	F	1	0
*Andrena (Simandrena) lepida*	M	0	1
*Andrena (Zonandrena) flavipes*	F	3	1
*Andrena (Zonandrena) flavipes*	M	0	3
*Anthophora (Pyganthophora) sichelii*	F	2	1
*Anthophora (Pyganthophora) sichelii*	M	2	19
*Apis mellifera*	F	16	10
*Apis mellifera*	M	0	4
*Colletes sp*.	F	2	2
*Merodon trochantericus*	F	1	0
*Merodon trochantericus*	M	0	1
*Eucera graeca*	F	2	2
*Eucera nigrescens*	F	3	1
*Eucera nigrescens*	M	0	9
*Eucera oraniensis*	F	1	7
*Eucera oraniensis*	M	2	6
*Eumenidae sp*.	F	2	2
*Lasioglossum sp*.	F	2	2
*Lasioglossum sp*.	M	0	4
*Megachile (Chalicodoma) sicula*	F	3	0
*Megachile (Chalicodoma) sicula*	M	0	7
*Melecta albifrons nigra*	F	1	0
*Melecta albifrons nigra*	M	0	1
*Osmia (Chalcosmia) caerulescens*	F	2	2
*Osmia (Chalcosmia) caerulescens*	M	0	2
*Osmia (Helicosmia) latreillei*	F	1	0
*Osmia (Helicosmia) latreillei*	M	0	1
*Osmia (Pyrosmia) ferruginea igneopurpurea*	F	2	0
*Osmia (Pyrosmia) ferruginea igneopurpurea*	M	0	1
*Osmia bicornis*	F	1	1
*Rhodanthidium sticticum*	F	3	2
*Rhodanthidium sticticum*	M	1	10
*Rhodanthidium sp*.	F	1	1
*Tetralonia sp*.	F	2	1
*Tetralonia sp*.	M	0	1
*Eucera sp*.	F	1	0
*Andrena sp. (A)*	F	2	2
*Andrena sp. (B)*	F	1	3
*Andrena sp. (C)*	M	0	9

**FIGURE 4:**
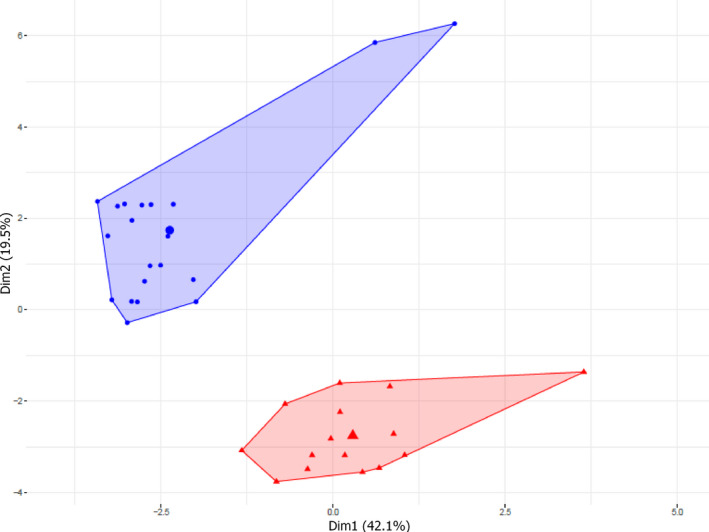
Differentiation in cuticular hydrocarbons between the two *Anacamptis papilionacea* ecotypes. Hierarchical Clustering on Principal Component (HCPC) between *A. p. papilionacea* and *A. p. grandiflora* based on the relative amount of 32 scent compounds. The cluster represented by blue circles includes all *A. p. papilionacea* individuals while the cluster represented by red triangles includes all *A. p. grandiflora* individuals

*A. p. papilionacea* and *A. p. grandiflora* differed in their floral cuticular hydrocarbons. Several compounds were exclusively present in one ecotype (*A. p. papilionacea*: C30, C33, (*Z*)‐7‐C33, (*Z*)‐9‐C23, (*Z*)‐11‐C33; *A. p. grandiflora*: C22, (*Z*)‐7‐C21, (*Z*)‐11‐C21, (*Z*)‐11‐C23, (*Z*)‐11‐C31). In terms of relative amounts, significant differences were found for many compounds present in both ecotypes (Table 4). In particular, large differences in relative amount between ecotypes were detected for C27, C28, and (*Z*)‐7‐C25. The HCPC analysis conducted on the relative concentration values showed a clear differentiation between *A. p. papilionacea* and *A. p. grandiflora* (Figure 4).

**TABLE 4:** Relative amounts of 32 cuticular hydrocarbons in *A. p. papilionacea* and *A. p. grandiflora*

	*A. p. papilionacea*	*A. p. grandiflora*	*F*	*p*
C33	7.507 (3.253)	0.000	101.434	**<0.001**
(*Z*)‐11‐C33	4.038 (1.085)	0.000	219.975	**<0.001**
(*Z*)‐7‐C33	7.267 (3.181)	0.000	89.83	**<0.001**
C31	8.195 (2.231)	8.054 (3.201)	0.085	0.772
*(Z*)‐11‐C31	0.000	1.361 (1.648)	15.309	**<0.001**
(*Z*)‐9‐C31	1.430 (1.382)	2.347 (1.789)	2.512	0.121
(*Z*)‐7‐C31	7.114 (2.673)	4.320 (2.757)	14.303	**0.001**
C30	2.859 (1.633)	0.000	68.904	**<0.001**
C29	14.495 (3.573)	14.545 (4.460)	0.001	0.981
(*Z*)‐11‐C29	1.618 (3.587)	2.074 (1.809)	0.117	0.734
(*Z*)‐9‐C29	0.100 (0.301)	3.560 (2.511)	44.503	**<0.001**
(*Z*)‐7‐C29	7.222 (2.622)	4.107 (3.257)	13.622	**0.001**
C28	7.659 (9.292)	1.509 (1.453)	10.387	**0.003**
C27	5.754 (1.797)	15.988 (6.139)	40.206	**<0.001**
(*Z*)‐11‐C27	1.158 (0.978)	2.189 (1.639)	5.412	0.025
(*Z*)‐9‐C27	0.108 (0.301)	2.106 (1.575)	37.655	**<0.001**
(Z)‐7‐C27	1.343 (1.187)	2.740 (1.951)	7.352	**0.01**
C26	0.969 (1.103)	2.202 (1.743)	6.549	0.015
C25	14.721 (4.042)	14.861 (5.624)	0.029	0.865
(*Z*)‐11‐C25	9.892 (4.583)	9.330 (4.550)	0.059	0.809
(*Z*)‐9‐C25	0.839 (0.638)	1.347 (1.262)	2.252	0.142
(*Z*)‐7‐C25	0.182 (0.556)	3.311 (3.533)	15.938	**<0.001**
C24	1.188 (1.065)	1.806 (1.766)	1.381	0.247
C23	6.153 (2.036)	6.907 (1.688)	1.042	0.314
(*Z*)‐11‐C23	0.000	1.265 (1.093)	32.95	**<0.001**
(*Z*)‐9‐C23	1.362 (1.227)	0.000	22.32	**<0.001**
(*Z*)‐7‐C23	0.400 (0.634)	1.995 (1.797)	14.433	**0.001**
C22	0.000	2.632 (1.806)	54.438	**<0.001**
C21	1.410 (1.364)	3.195 (1.192)	20.273	**<0.001**
(*Z*)‐11‐C21	0.000	1.471 (1.752)	15.906	**<0.001**
(*Z*)‐7‐C21	0.000	1.961 (2.544)	11.286	**0.002**
C19	1.127 (0.941)	1.590 (1.489)	0.816	0.372

In bold significant differences after one‐way ANOVA.

In the sympatric population of San Priamo, pollen transfer efficiency differed for the two ecotypes. In *A. p. papilionacea*, among the 180 observed flowers (from 21 individuals) 80 were visited (i.e., with pollinia removed) and 33 were pollinated (pollen transfer efficiency = 0.41 ± 0.0724); in *A. p. grandiflora*, among the 49 observed flowers (from 9 individuals), 33 were visited (i.e., with pollinia removed) and 8 were pollinated (pollen transfer efficiency = 0.20 ± 0.0851).

### 3.4 Environmental niche modeling (ENM) and estimation of ecogeographic isolation index

**FIGURE 5:**
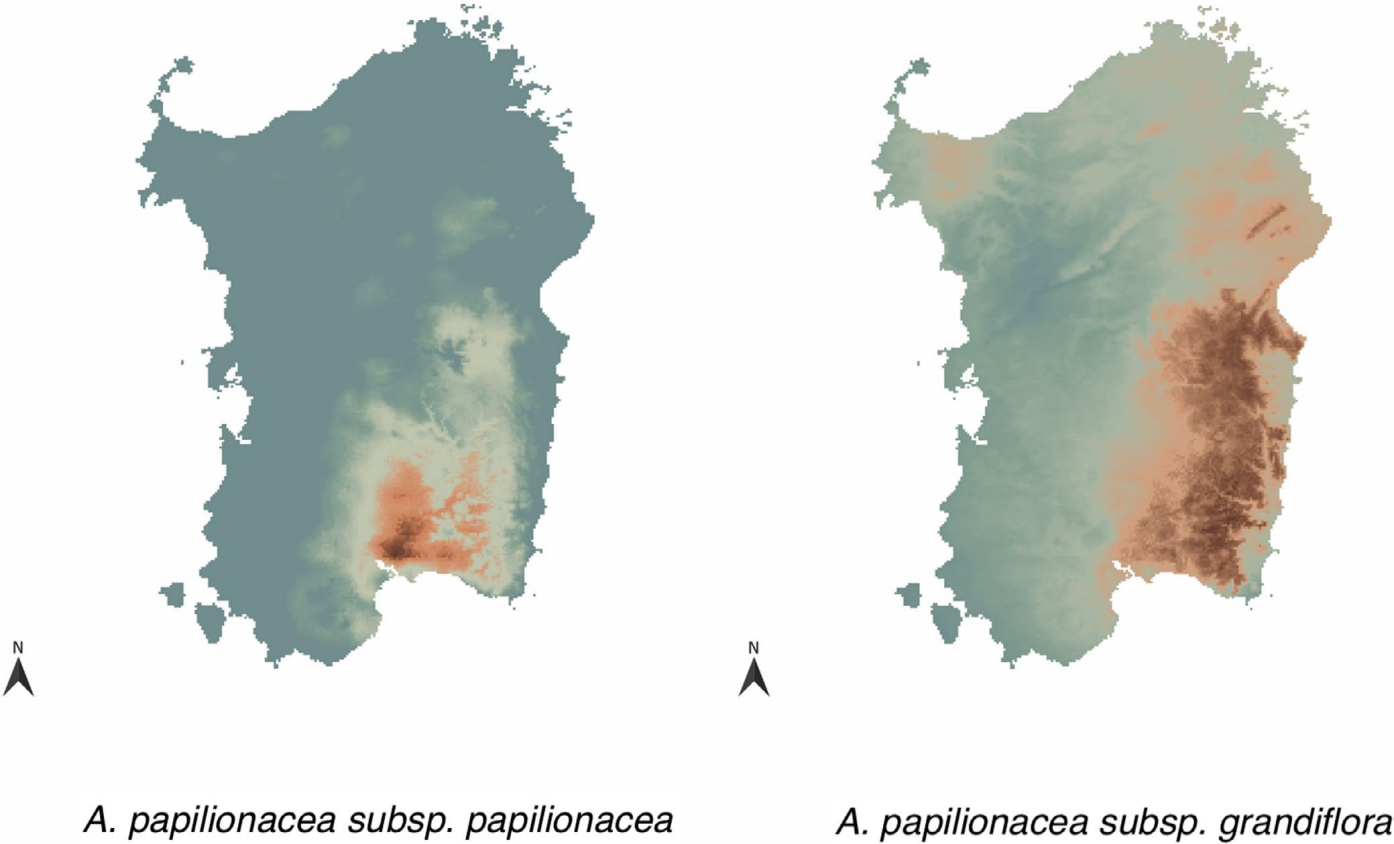
Ecogeographic niches of the two *Anacamptis papilionacea* ecotypes. Environmental niche analysis estimated using Maxent and showing predicted niches for *A. p. papilionacea* (a) and for *A. p. grandiflora* (b) on Sardinia

Among the seven selected climatic variables, temperature seasonality and precipitation seasonality were the more important variables for describing *A. p. papilionacea* and *A. p. grandiflora* ecological niches. Mean value and coefficient of variation (CV) for the seven climatic variables used in the model are reported in Table 1. The two ecotypes show different ecological preferences with the niche of *A. p. papilionacea* being included in that of *A. p. grandiflora* (Figure 5). RI_ecogeography_, an index which ranges between 0 (no isolation) and 1 (complete isolation), was 0.81 in *A. p. grandiflora* and 0.25 in *A. p. papilionacea* (Table 5).

**TABLE 5:** Reproductive isolation indices

	RI ecogeography	RI phenology	RI floral isolation	RI postm_prez	RI postm_postz
*A. p. papilionacea*	0.25	0.07	0.62	0.00	0.00
*A. p. grandiflora*	0.81	0.08	0.71	0.00	−0.05

### 3.5 Flowering time estimation and calculation of phenological isolation index (RI _phenology_)

**FIGURE 6:**
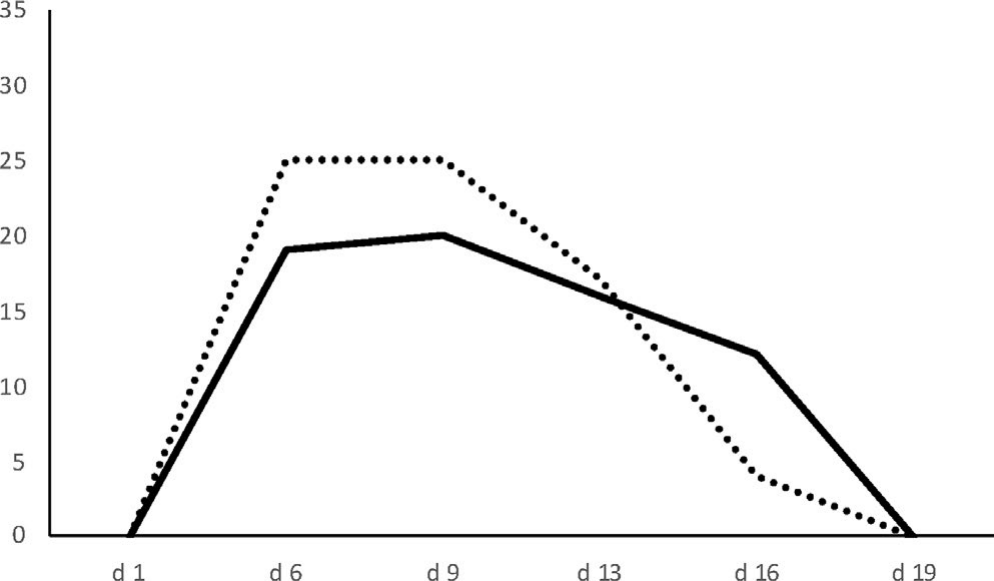
Flowering phenology overlap between the two *Anacamptis papilionacea* ecotypes. Phenology was estimated in naturally occurring *A. p. papilionacea* and *A. p. grandiflora* at sympatric site. Y‐axis: number of flowering individuals throughout the census period. X‐axis: sampling days. *A. p. papilionacea* dotted line, *A. p. grandiflora* continuous line

*A. p. papilionacea* and *A. p. grandiflora* show slightly different flowering times with *A. p. grandiflora* flowering earlier than *A. p. papilionacea* (Figure 6). *RI _phenology_* was 0.07 in *A. p. papilionacea* and 0.08 in *A. p. grandiflora*, respectively (Table 5).

### 3.6 Floral isolation

Experimental plots with stained pollen staining built in the two years showed similar results with a higher proportion of intra‐ than interecotype movements (80.2% in 2011 and 75.6% in 2017, respectively; see Table 6). Therefore, we found nonrandom mating and some degree of floral isolation between ecotypes (0.62 in *A. p. papilionacea* and 0.71 in *A. p. grandiflora*, respectively; Table 5).

**TABLE 6:** Intra‐ and interecotype pollen movements in experimental plots

Ecotype	Pollen movement	2011	2017
*A. p. grandiflora*	intraecotype	37	13
*A. p. grandiflora*	interecotype	4	3
*A. p. papilionacea*	intraecotype	125	28
*A. p. papilionacea*	interecotype	28	7

### 3.7 Postmating isolation

Percentage of fruit production was equal in intra‐ and interecotype crosses with the same ovule parent (100% on *A. p. papilionacea* and 83.3% on *A. p. grandiflora*, respectively). Thus, RI_postm_prezygotic_ was 0 in both *A. p. papilionacea* and *A. p. grandiflora*. No significant differences were found in the amount of viable seeds between the different crosses types (one‐way ANOVA: *F*_68.32,18_ = 0.17, *p* =0.92). Intraecotype crosses yielded an average seed set of 69.0 ± 18% for *A. p. grandiflora* and 66.2 ± 21% for *A. p. papilionacea*. Interecotype crosses yielded seed sets of 66.5 ± 22% when *A. p. grandiflora* was the pollen donor, and 77.0 ± 4% when *A. p. papilionacea* was the pollen donor. RI _embryo_mortality_ was thus very low in both ecotypes (0.002 in *A. p. papilionacea* and −0.05 in *A. p. grandiflora*; Table 5).

## 4 DISCUSSION

In this study, we investigated the mechanisms that allow coexistence of two ecotypes (*A. p. papilionacea* and *A. p. grandiflora*) of the circum‐Mediterranean orchid species *Anacamptis papilionacea*. Overall, as expected for ecotypes of one species, postmating barriers were very weak or absent between ecotypes. However, *A. p. papilionacea* and *A. p. grandiflora* showed significantly different geographic distribution and pollination mode, with a distinct prevalence of male pollinators found in *A. p. papilionacea*. These differences are thus considered key in maintaining ecogeographic and floral isolation between the ecotypes and may represent the initial step toward evolution of sexual mimicry within an orchid lineage with food deception as plesiomorphic pollination system. Still, such difference in geographic distribution and pollination mode is only an initial stage of ecological divergence between the two ecotypes, nor we cannot predict if it can move forward.

Ecogeographic isolation is often the earliest reproductive barrier arising among plant ecotypes and incipient species (Sobel, 2014). The geographic distribution of the two ecotypes in Sardinia reflects their different ecological habitat preferences and build up a strong but highly asymmetric ecogeographic barrier (Table 5). *A. p. papilionacea* appears to be more strictly linked to a Mediterranean climate (Figure 5), with a distribution influenced by temperature and precipitation seasonality, while *A. p. grandiflora* has a wider altitudinal range, occurring up to 2000m altitude, and a broader niche on Sardinia. These differences in ecogeographic preferences can slow down intermixing of the two ecotypes because geographic partition can decrease the chance of random mating. However, this mechanism appears to contribute more in *A. p. grandiflora* that has a more exclusive distribution, rather than in *A. p. papilionacea* whose distribution is almost included within that of *A. p. grandiflora*. Nevertheless, microhabitat preference of ecotypes (not estimated here) can still contribute to strength the ecogeographical isolation at local scale.

In the Mediterranean basin, the frequent changes of land connection and insularity due to geological events and the habitat fragmentations due to intense anthropogenic pressures promote the frequent occurrence of secondary contact of allopatrically diverged lineages (Feliner, 2014; Pavarese et al., 2011; Zitari et al., 2011). Thus, despite different ecological preferences, *A. p. papilionacea* and *A. p. grandiflora* ecotypes often coexist in Sardinia. In contrast to the admixture found in other contact zones between ecotypes/vicariant species of food‐deceptive orchids (Zitari et al., 2012), we found that *A. p. papilionacea* and *A. p. grandiflora* ecotypes are phenotypically differentiated both in the allopatric and, more importantly, in the sympatric populations (Figure 2). Persistence of phenotypic divergence even in sympatric spots, even if we cannot fully exclude some genetic admixture, suggests that some isolating barriers should exist between the two ecotypes. In absence of postmating isolation, floral isolation, with *A. p. papilionacea* mainly attracting male pollinators, while *A. p. grandiflora* mainly attracting female pollinators seems the main premating barrier. Given male pre‐emergence is known for many solitary bees, a potential pollinator sex bias (in terms of local pollinator availability) cannot be excluded in allopatric populations in spite of their geographic proximity and overlapping phenology. Such difference in pollinator attraction (and morphology), however, also occurs in the sympatric populations where the two ecotypes are certainly exposed to the same pollinator regime. Usually, phenological or floral isolation is weak in generalized food‐deceptive orchids species pairs because they attract a wide range of different pollinators and thus have a high chance of pollinator overlap and low selection for flowering time (Cozzolino et al., 2005). In that specific case, we argue that the two ecotypes achieve floral isolation by adopting two partly different pollination strategies. This difference is mirrored by the difference found in pollen transfer efficiency in the sympatric population of San Priamo under a common pollinator regime. Our result concords with previous records of pollen transfer efficiency for allopatric *A. p. papilionacea* and *A. p. grandiflora* (Scopece et al., 2015), being higher in the former than in the latter ecotype. Difference in pollen transfer efficiency was found to be strictly linked to the exploitation of different pollination strategies, with sexually deceptive species experiencing higher values than food‐deceptive species (Scopece et al., 2010).

The two ecotypes also differ in floral hydrocarbon bouquets (Figure 4) both in terms of presence/absence of specific compounds and in terms of relative amounts of other compounds (Table 4). These hydrocarbons (Mant et al., 2005) and other types of compounds (as polar compounds, Cuervo et al., 2017) are often used by the closely related sexually deceptive *Ophrys* species for attract their specific male pollinators (Ayasse et al., 2011; Schiestl et al., 2000).

Attraction of male pollinators may be initially facilitated by olfactory signals and only secondarily by morphological adaptation of the flower. There are several cases of orchids, as for instance *Disa atricapilla* and *Disa bivalvata*, that look like food‐deceptive orchids, yet sexually attract male pollinators by emissions of specific scent bouquets (Steiner et al., 1994). In other examples, such as in *Orchis galilaea*, which shares flower shape and color with its allied food‐deceptive sister species, only males *Halictus* bees were found as pollinators (Bino et al., 1982). As hypothesized by Schiestl and Cozzolino (2008), the widespread occurrence of unsaturated hydrocarbons (alkenes) which have a fundamental role in sexual mimicry in the genus *Ophrys*, may be an exaptation for the evolution of “incipient” sexual deception (sensu Johnson & Schiestl, 2016). Electro‐antennographic detection (GC–EAD) studies and behavioral assays are needed (Schiestl & Marion‐Pol, 2002) for demonstrating whether some olfactory cues play a similarly important role in *A. p. papilionacea* as found in sexually true deceptive orchids such as the genus *Ophrys* (Ayasse et al., 2011; Schiestl et al., 2000). As an alternative hypothesis, scent differences between *A. p. papilionacea* and *A. p. grandiflora* may be nonadaptive and just the consequence of different patterns of genetic drift or trait correlations (Juillet et al., 2011).

Our pollen staining experiment showed that attraction of different pollinators could lead to floral isolation between the two ecotypes. Indeed, we found that most of pollen movements (around 80%) were intraecotype. In our study system, thus, floral isolation is likely to be the main isolating mechanism between ecotypes in sympatric populations. Nevertheless, even if ecogeographic isolation (mainly for *A. p. grandiflora*) and floral isolation (for both ecotypes) certainly contributed to the isolation of the two ecotypes, the amount of interecotype pollen movements (about 20%) in sympatry is still not negligible and likely sufficient to lead to a quick genetic homogenization of the allopatrically gained phenotypic divergence. At the same time differences in flower phenology were very small in sympatry (Figure 6) thus the question of why the two ecotypes do not merge and produce intermediate phenotypes in face of a significant residual gene flow remains open. Because we did not observe intermediate phenotypes in the field despite the potential for residual gene flow in sympatry, we may speculate that the genetic architecture of phenotypic differences between the ecotypes may allow some degree of interbreeding without producing intermediate phenotypes (Scopece et al., 2020). A strong linkage of floral traits determining pollination syndromes, as already found in other plant systems (Hermann et al., 2013; Zu et al., 2016), and/or the presence of a master gene/supergene (i.e., as those controlling local mimicry polymorphism in butterflies, Le Poul et al., 2014) with a dominance phenotype determining both the flower and pollination type, may be the underlying reason for distinct phenotypes despite gene flow between *A. p. papilionacea* and *A. p. grandiflora*. In that case, the frequency of the dominant allele/linkage group for determining both the flower and pollination type can be subject to local selection by prevalent pollinator community (Kellenberger et al., 2019) so determining the different ecogeographic distribution and the relative abundance of *A. p. papilionacea* and *A. p. grandiflora* in the contact zones.

## CONFLICT OF INTEREST

The authors of this manuscript declare no conflict of interest, financial, or otherwise.

## AUTHOR CONTRIBUTION

**Salvatore Cozzolino**: Conceptualization (lead); Writing‐original draft (equal). **Giovanni Scopece**: Conceptualization (equal); Investigation (equal); Writing‐original draft (equal). **Michele Lussu**: Data curation (equal); Formal analysis (equal); Investigation (equal). **Pierluigi Cortis**: Data curation (equal); Formal analysis (equal). **Florian P. Schiestl**: Conceptualization (equal); Funding acquisition (lead); Writing‐review & editing (equal).

## Data Availability

Dataset on morphometry is available at: https://doi.org/10.5061/dryad.m905qfv0p
